# Psychometric properties of the Chinese version of the instrument for measuring different types of cognitive load (MDT‐CL)

**DOI:** 10.1111/jonm.12919

**Published:** 2020-03-28

**Authors:** Shan Zhang, Ying Wu, Ziyuan Fu, Yating Lu, Qingyu Wang, Liu Mingxuan

**Affiliations:** ^1^ School of Nursing Capital Medical University Beijing China

**Keywords:** Chinese, instrument for measuring different types of cognitive load, nurse, reliability, validity

## Abstract

**Aim:**

To translate the instrument for measuring different types of cognitive load (MDT‐CL) into Chinese and assess the reliability and validity of the Chinese version of the MDT‐CL.

**Background:**

The MDT‐CL is needed for hospital administrators to identify which nursing staff are prone to high cognitive load and to provide tailored interventions for specific types of cognitive load.

**Methods:**

The MDT‐CL was translated into Chinese using forward and back translation, cultural adaptation and pilot tested. The reliability and validity of the instrument were assessed with intensive care unit (ICU) nurses in three tertiary hospitals in China.

**Results:**

A total of 222 ICU nurses were recruited. The scale‐content validity index of the Chinese version of the MDT‐CL was 0.966. Confirmatory factor analysis indicated that all the goodness‐of‐fit indicators were acceptable. Cronbach's α coefficient was 0.818. Test–retest reliability was 0.785.

**Conclusions:**

The Chinese version of the MDT‐CL is a valid and reliable instrument for evaluating the cognitive load of ICU nurses in China.

**Implications for nursing management:**

The validated Chinese version of the MDT‐CL is a feasible, quantitative tool for evaluating different types of cognitive load in busy clinical practice, suggesting significant clinical application value.

## 1 INTRODUCTION

Cognitive load (CL) refers to the total amount of cognitive resources that a person needs to process cognitive activities, and the capacity of cognitive resources is limited while executing complex activities, with a particular focus on maximal cognitive resources (Sweller, 1988; Sweller, Van, & Paas, 1998). Appropriate CL has been identified as very important for occupations that require extensive use of cognitive resources to perform the work efficiently (Mohammadi, Mazloumi, Kazemi, & Zeraati, 2015; Pawar, Jacques, Deshpande, Pusapati, & Meguerdichian, 2018). Cognitive overloads, which exceeds one's cognitive resource capacity, are often related to lower speed of receiving and processing information, decreased capacity of cognitive resources and poor performance during work (Kirschner, Sweller, Kirschner, & Zambrano, 2018; Vogels, Demberg, & Kray, 2018; Watanabe & Yamauchi, 2018).

Nursing care in the intensive care units (ICU) is characterized by extremely demanding caseloads, performance of complicated activities and making complex decisions (Mohammadi et al., 2015), leading to high CL of ICU nurses. One example is the complex care required in ICU delirium prevention and management interventions. ICU delirium is a common complication of ICU patients with a high incidence of 70% to 87% (Klein et al., 2014) and associated with longer hospital length of stay (LOS) and increased mortality (Barnes‐Daly, Phillips, & Ely, 2017; Devlin et al., 2018). Guidelines recommend use of the bundle intervention as a way to prevent and manage ICU delirium (Devlin et al., 2018). However, adherence to the delirium bundle interventions is suboptimal in routine clinical care, and the mainly possible reason is that the bundle intervention can cause high CL (Morandi et al., 2017). Therefore, we chose delirium care rather than other aspect of patient care to study the CL.

Higher CL is a major concern reported by ICU nurses in clinical care, which negatively impacts nurses and their patients (Mohammadi et al., 2015). High CL, which can lead to psychological distress and mental fatigue (Duhoux, Menear, Charron, Lavoie‐Tremblay, & Alderson, 2017; Sonmez, Oguz, Kutlu, & Yildirim, 2017; Wheelock et al., 2015), is detrimental to the performance of activities (Watanabe & Yamauchi, 2018), impedes opportunities for acquiring knowledge (Song et al., 2014; Starmer et al., 2014), results in poor patient outcomes and compromises patient safety (Watanabe & Yamauchi, 2018; Wheelock et al., 2015).

Considering the negative consequence of high CL, it is important to assess different types of CL and to provide tailored interventions for reducing the CL (Duhoux et al., 2017). The assessment methods of CL have been separated into physiological, and dual‐task paradigm and psychometric measures (Naismith & Cavalcanti, 2015). Physiological parameters (e.g. heart rate variability, electroencephalography) are affected by many factors (e.g. emotions, workload); therefore, physiological parameters’ results are difficult to measure accurately (Naismith & Cavalcanti, 2015). Additionally, dual‐task paradigm parameters are often assessed in a laboratory and therefore not practical for routine use in the clinical practice. Whereas psychometric scales are less expensive and simpler to use, subjective assessment is still the most commonly used method to measure CL (Naismith & Cavalcanti, 2015; Sonmez et al., 2017), such as Paas's self‐rating scale (Paas, Merrienboer, & Adam, 1994) and the National Aeronautics and Space Administration‐Task Index (NASA‐TLX) (Hart & Staveland 1988; Paas et al., 1994; Sonmez et al., 2017). These assessment tools are used to measure overall CL, and they do not effectively distinguish different types of CL (Leppink, Paas, Vleuten, Gog, & Merrienboer, 2013). Cognitive load is composed of three domains: intrinsic CL, extraneous CL and germane CL (Paas et al., 1994). *Intrinsic CL* is generated by the information elements in cognitive activities and their interaction during cognitive processing (Scheiter, Gerjets, & Catrambone, 2006) and can be affected by the difficulty of a given activity and the prior knowledge of the activity performer (Hsieh, Hsu, & Huang, 2016). *Extraneous CL* refers to the cognitive resource allocated to deal with irrelevant cognitive activities, which is caused by suboptimal instructional methods, the unclear instruction will interfere activities completion (Scheiter et al., 2006). *Germane CL* involves storing new information in long‐term memory, which allows for individuals to concentrate more on execution of activities (Sweller et al., 1998). Therefore, an instrument which can measure the three different types of CL is needed to provide targeted interventions for the specific types of CL and hence improve the CL of the study subjects.

Leppink and colleagues (Leppink et al., 2013) developed a ten‐item scale for measuring different types of cognitive load (MDT‐CL), a three‐component scale was established by principal component analysis and confirmed by a confirmatory factor analysis (CFA) of data from three lectures in statistics that focused on learning the topic, formulas, concepts and definitions, and the English version of the MDT‐CL has a simple structure and a high reliability and validity (Leppink et al., 2013). To our knowledge, this tool has not been translated into Chinese and validated in a Chinese population, which is important given the cultural differences between Western countries and China. Since the MDT‐CL scale is not limited to a specific knowledge domain, it can be modified and replaced according to the researcher's focus of study and task presentation method. Therefore, in our study each item revolved around daily care for patients with ICU delirium and focused on the CL of ICU nurses during their daily usual care in relation to ICU delirium. Thus, the aim of this study was to translate the MDT‐CL into Chinese and assess the reliability and validity of the MDT‐CL with nursing staff in China in order to provide a reliable instrument to effectively evaluate the CL of nurses in health care institutions.

## 2 MATERIALS AND METHODS

This study was conducted in two phases: translation (forward and back translation, cultural adaptation and pilot testing) and a cross‐sectional study to validate the scale in a Chinese nursing population.

## 3 TRANSLATION PROCEDURE

### 3.1 Forward and back translation

The authors contacted Dr. Leppink by email to obtain permission to translate the original MDT‐CL into Chinese. We conducted translation according to the translation model proposed by Brislin (1970) for maintaining both conceptual and linguistic equivalence: forward and back translation, cultural adaptation and pilot testing (Brislin, 1970). First, two native Chinese‐speaking nursing students (one PhD student and one master degree student) who are proficient in English translated the English version MDT‐CL into a Chinese version A and B independently, and the research team discussed and modified discrepancies items between the two forward‐translated versions and integrated these changes into the Chinese version C. Second, back translation of the Chinese version C was conducted by two bilingual nurse educators who were not aware of the original English version. The research team compared the back translation version and original English version to make appropriate changes on the Chinese version C and then repeated translation and back translation until back translation with the original MDT‐CL to achieved high consistency; ultimately, the Chinese version D of MDT‐CL was formed.

### 3.2 Cultural adaptation

To confirm that the concepts and connotation of each item were kept, a panel of six experts (one psychological expert, two clinical nursing experts, two delirium clinician experts and one English teacher) and all translators compared the differences between the original English scale and the Chinese version D, including linguistic and semantic congruence, cultural relevancy and conceptual equivalences. The Chinese version D of MDT‐CL was then modified according to the different knowledge domain, study participators and settings to develop the Chinese version E.

### 3.3 Pilot testing

A pilot study was conducted with 30 ICU nurses to ensure that the Chinese version E was suitable for the use of clinical nursing staff, including the nurses' perception of the simplicity and content understanding of the scale. In addition, the time spent completing the scale was recorded and suggestions for improving each item were collected. Based on feedback from the nurses, appropriate modifications were made to the Chinese version E, leading to the development of Chinese version of the MDT‐CL.

### 3.4 Reliability and validity testing of the Chinese version MDT‐CL

#### 3.4.1 Study design and setting

A cross‐sectional study was conducted to validate the Chinese version MDT‐CL in three tertiary hospitals. A convenience sample of registered nurses who worked in ICU settings was recruited from three large university‐affiliated hospitals in Beijing between October 2018 and March 2019.

#### 3.4.2 Participants

Nurses were eligible for the study if they (a) had a minimum of one‐year experience in intensive care; (b) worked full time in the unit; and (c) consented to participate in this study. Nurses who were on leave for various reasons during the study period were excluded. The rule of thumb for calculating the sample size of reliability and validity test of the scale is that at least 10 subjects should be allocated for each item (Wilson, Voorhis, & Morgan, 2007). The Chinese version MDT‐CL is composed of 10 items. In our study, 20 participants were chosen for each item. In total, 200 participants were needed. Considering invalid questionnaires, the sample size was expanded by 10%, and therefore, 222 participants were required.

#### 3.4.3 Measures

##### The Chinese version of the MDT‐CL

The MDT‐CL consists of ten items with three subscales: intrinsic CL (items 1, 2 and 3), extraneous CL (items 4, 5 and 6) and germane CL (items 7, 8, 9 and 10). Each item is scored from 0 to 10 with anchor words (e.g. not at all, completely), the higher the score, the higher the CL.

Demographic information collected included the following: age, gender, level of education, professional title, years of ICU experience and departments.

##### The National aeronautics and space administration‐task load index (NASA‐TLX)

To determine criteria validity, we compared the MDT‐CL with the NASA‐TLX, which is widely used to assess CL and consists of six dimensions (mental demand, physical demand, temporal demand, effort demand, performance and frustration demand), and each dimension is scored from 0 to 20, the lower the score, the lower the CL (Hart & Staveland, 1988). Content validity in the Chinese version of NASA‐TLX was 0.900, and Cronbach's α values were 0.784, a good consistency between each item and the overall scale (Xiao, Wang, Wang, & Lan, 2005).

##### Paas's (1992) nine‐point scale

Paas's scale is widely used to measure the degree of mental effort used when completing an activity and is considered as a reliable estimator of overall CL (Ayres, 2006; Paas, Renkl, & Sweller, 2003).

##### Measurements for the three different types of CL

A nine‐point Likert scale was used from 1 (extremely little/low/easy) to 9 (extremely much/high/difficult). (a)To measure intrinsic CL, a single item was adapted from Ayres (Ayres, 2006) and asked “How difficult do you think delirium care activities are?”. (b)To measure extraneous CL, a single item was adapted from Cierniak et al. (Cierniak, Scheiter, & Gerjets, 2009) and asked “How difficult were the instructions for delirium care?”. (c)To measure germane CL, a single item was adapted from Salomon (Salomon, 1984) and asked “How much do you concentrate when providing delirium care?”. The three single‐item tools were highly reliable (Ayres, 2006; Cierniak et al., 2009; Salomon, 1984) and also used in the Leppink study as criteria instruments (Leppink et al., 2013).

#### 3.4.4 Validity and reliability

Content validity, construct validity and criterion validity were used to evaluate the validity of the scale. Content validity was based on expert consultation (Hair, Black, Babin, Anderson, & Tatham, 2006). The inclusion criteria for the experts included bachelor's degree or above and more than 10‐year work experience. Reliability is generally evaluated by Cronbach's α coefficient and test–retest reliability. The sample size of the test–retest reliability was 4 times the number of items (Park, Kang, Jang, Lee, & Chang, 2018). Two weeks later after the initial test, 40 randomly selected nurses from the first test nurses were asked to complete the same scale for the second time.

#### 3.4.5 Data collection

Before study commencement, support for this study was obtained from all participating hospitals and nursing supervisors. Convenience sampling was used to recruit eligible ICU nurses. The purpose and significance of the study, the instrument, and also the anonymity and confidentiality of the data were fully explained to all eligible participants. Informed consent was obtained verbally from all subjects prior to completing the questionnaire. Participation was voluntary, and participants were told that they could withdraw from the study at any time without consequences. During completion of the questionnaire, the investigators were available to answer any questions related to the survey.

#### 3.4.6 Data analysis

The SPSS version 21.0 (SPSS Inc.) was used for data analysis, and AMOS 21.0 statistical software for structural equation modelling (*SEM*) was used to explain the relationship among variables. Continuous variables were described as means and standard deviation (*SD*) for normal distributed data and medians and interquartile range otherwise. Categorical variables were expressed as frequencies or percentages.

Content validity of the scale was determined with a content validity index (CVI), based on expert consultation (Hair et al., 2006). Confirmatory factor analysis (CFA) was adopted to assess the construct validity. The following criteria were used to evaluate model goodness‐of‐fit: goodness‐of‐fit index (GFI) >0.85, comparative fit index (CFI) >0.90 and Tucker–Lewis Index (TLI) >0.90, and root‐mean‐square error of approximation (RMSEA) <0.05 indicated that the model was accepted (Bentler, 1990; Brown, 2006). Chi‐squared/degrees of freedom (df) ratio (*χ^2^/*df) between 1 and 5 indicates a good global fit (Olson, Hayduk, & Thomas, 2014). Spearman correlation analysis was used for criterion validity and inter‐correlations between the items, the factors and the total scale. The internal consistency of the scale was determined by Cronbach's α coefficient, with α value of the total scale better than 0.8 is considered good reliability (Terwee et al., 2007). Paired *t* tests were used to assess the relationship and correlation coefficient (*r*) between the test and retest scores, with *r* > 0.7 considered good stability and consistency. All tests were two‐tailed, and *p*‐value < .05 was considered statistically significant.

## 4 RESULTS

### 4.1 Cross‐cultural translation and adaptation

The English version MDT‐CL was successfully translated into Chinese, and all nurses reported that they were able to answer the questions without any difficulties. However, minor modifications in wording were made based on different knowledge domains (Table 1). For example, “formulas” in items 2 and 9 were changed to “delirium prevention or management interventions.”

**Table 1:** Modifications of cross‐cultural adaptation

items	Original scale	Chinese version scale
1, 2, 3, 4, 7, 8, 9, 10	Activity	Daily usual care
1, 7	Topic/topics	Delirium care activity/activities
2, 9	Formulas	Delirium prevention or management interventions
3, 10	Concepts and definitions	Delirium and risk factors assessment
5	Learning	Clinical application
8	Statistics	Delirium

### 4.2 Content validity

Six experts were invited to rate content relevance on each item based on a 4‐point Likert scale: 1 = not relevant, 2 = weak relevance, 3 = relevant and 4 = strong relevance. Five females (83.33%) participated in the process. Mean age was 45.17 ± 7.08 years. Mean work experience was 22.67 ± 7.39 years. Three experts had doctoral degrees (50.00%) and the others master's degrees (50.00%). The item‐level content validity index (I‐CVI) was 0.83 ~ 1.00, and the scale‐level content validity index (S‐CVI) was 0.966.

### 4.3 Sample characteristics

A total of 222 questionnaires were administered, and 12 were invalid due to missing data. The response rate was 94.59% (210/222). A total of 200 (95.24%) were female. Mean age was 30.17 ± 4.27 (range, 21–45) years old. Mean years of ICU experience was 7.36 ± 4.61 (range, 1–26) years (Table 2).

**Table 2:** Characteristics of study subjects

Characteristics	Pilot study	Reliability and validity
	*N* = 30	*N* = 210
Age (years)	33.37 ± 4.58	30.17 ± 4.27
Gender *n* (%)		
Male	3 (10.00%)	10 (4.76%)
Female	27 (90.00%)	200 (95.24%)
Ethnicity *n* (%)		
Han	30 (100.00%)	205 (97.62%)
Mongolian	0 (0.00%)	2 (0.48%)
Manchu	0 (0.00%)	1 (0.95%)
Hui	0 (0.00%)	2 (0.95%)
ICU experience (years)	11.43 ± 4.98	7.36 ± 4.61
Marital status *n* (%)		
Married	24 (80.00%)	129 (61.43)
Single	6 (20.00%)	78 (37.14)
Divorced	0 (0.00%)	3 (1.43%)
Average family income (yuan) *n* (%)		
<3,000	0 (0.00%)	9 (4.29%)
3,000 ~ 8,000	9 (30.00%)	109 (51.90%)
>8,000	21 (70.00%)	92 (43.81)
Education level *n* (%)		
<High school	0 (0.00%)	2 (0.95%)
High school	12 (40.00%)	55 (26.19%)
Bachelor degree	17 (56.67%)	151 (71.90%)
Master degree	1 (33.33%)	2 (0.95%)
Departments *n* (%)		
CICU†	10 (33.33%)	51 (24.29%)
RCU‡	10 (33.33%)	41 (19.52%)
SICU§	10 (33.34%)	83 (39.52%)
NICU¶	0 (0.00%)	35 (16.67%)
Professional Title *n* (%)		
Primary nurse aide	4 (13.33%)	48 (22.86%)
Senior nurse	18 (60.00%)	146 (69.52%)
Supervisor nurse	8 (26.67%)	16 (7.62%)
Average number of patients cared for per day	2.93 ± 1.05	2.89 ± 0.89

^†^ Cardiac intensive care unit

^‡^ respiratory intensive care unit

^§^ surgical intensive care unit

^¶^ neurology intensive care unit.

### 4.4 Construct validity

**Figure 1:**
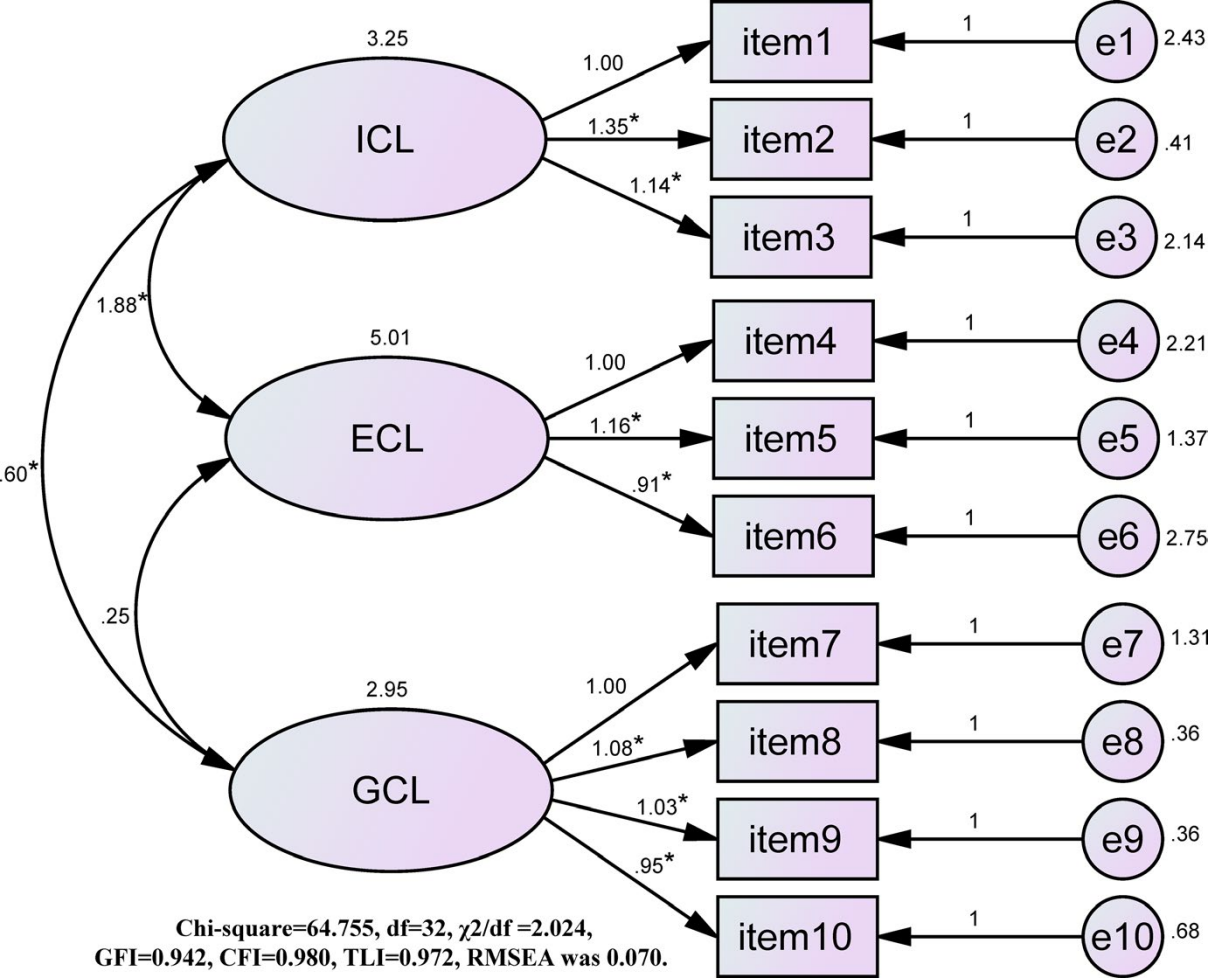
Three‐factor model of the instrument for measuring different types of cognitive load. ICL, intrinsic cognitive load; ECL, extraneous cognitive load; GCL, germane cognitive load; e, estimate. *p < .05 [Colour figure can be viewed at http://wileyonlinelibrary.com]

Exploratory factor analysis (EFA) revealed a three‐factor model, which showed consistent constructs with the original study, so only CFA is reported here (Figure 1). CFA of a three‐factor model was performed on 210 valid questionnaires and identified that *χ^2^/df* was 2.024 (<3.00), GFI was 0.942 (>0.85), CFI was 0.980 (>0.90), TLI was 0.972 (>0.90), and RMSEA was 0.070 (≤0.08). No model modification was made.

### 4.5 Criterion validity

The Spearman correlation analysis revealed that the correlation coefficient between NASA‐TLX and the Chinese version MDT‐CL was 0.911 (*p* < .01). The correlation coefficients between NASA‐TLX and intrinsic, extraneous and germane CL were 0.712, 0.495 and 0.564, respectively (*p* < .01). In addition, the scores between the intrinsic CL factor and Ayres's single item were positively correlated (*r* = 0.772, *p* < .01). Similar results were also found between extraneous CL factor and Cierniak's single item (*r* = 0.890, *p* < .01); germane CL factor and Salomon's single item (*r* = 0.747, *p* < .01); and overall CL and Paas's single item (*r* = 0.762, *p* < .01).

### 4.6 Internal consistency

Cronbach's α values of the overall Chinese version MDT‐CL was 0.818, and Cronbach's α values for the intrinsic, extraneous and germane CL were 0.879, 0.878 and 0.946, respectively. Table 3 shows the correlations among the three factors and the total scale. The correlation coefficient between each subscale was 0.003 ~ 0.367. Spearman's correlation coefficient between each item and the overall scale ranged from 0.490 to 0.755 (*p* < .01).

**Table 3:** Spearman's correlations between the subscale factors representing MDT‐CL and the total scale

Factor	Total	GCL†	ICL‡	ECL§
Total	1			
GCL†	0.604*	1		
ICL‡	0.791*	0.317*	1	
ECL§	0.573*	0.003	0.367	1

* correlations are significant at the 0.01 level (two‐tailed).

^†^ germane cognitive load.

^‡^ intrinsic cognitive load.

^§^ extraneous cognitive load.

### 4.7 Test–retest reliability

There was no statistically significant difference in intrinsic CL (*p* = .645), germane CL (*p* = .120) and overall CL (*p* = .161) between test and retest scores, and only extraneous CL (*p* = .011) between the two tests revealed differences. The intraclass correlation coefficient of the Chinese MDT‐CL after the two‐week interval for the repeated testing was 0.785 (*p* < .01, Table 4).

**Table 4:** Test–retest correlations of the MDT‐CL (*n* = 40)

Variables	Test		Retest		R
	Mean	*SD*	Mean	*SD*	
ICL†	6.66	2.29	6.78	1.63	0.718*
ECL‡	3.71	2.17	4.30	1.42	0.777*
GCL§	7.27	1.38	7.14	1.10	0.944*
MDT‐CL¶	6.02	1.16	6.18	0.87	0.785*

^†^ intrinsic cognitive load

^‡^ extraneous cognitive load

^§^ germane cognitive load

^¶^ an instrument for measuring different types of cognitive load.

* *p* < .01

## 5 DISCUSSION

To the best of our knowledge, this is the first study to evaluate CL of delirium care using the MDT‐CL in Chinese hospitals and to report the psychometric properties of this scale. This study serves to offer an assessment instrument to measure perceived CL in a large sample of 222 Chinese ICU nurses in three hospitals. The results of this study demonstrate that the MDT‐CL has satisfactory reliability and validity among ICU nurses. Most importantly, all participants perceived that the scale was user‐friendly, the questions were easy to comprehend, and it only took 3 to 5 minutes to complete the scale. Thus, the MDT‐CL is as informative as other indices for CL and provides a feasible, quantitative tool for busy clinical practice, suggesting significant clinical application value.

The authors made appropriate modifications according to the suggestions from the experts to form the final Chinese version MDT‐CL. Our results support that the Chinese version scale is appropriate and acceptable for use in settings in China, with good content validity (Palese et al., 2016).

The construct validity of the Chinese version MDT‐CL was examined with CFA, to confirm whether there was an adequate good fit between the hypothesized three‐factor model of the Chinese version MDT‐CL and the data. Prior to CFA, EFA was used to assess whether the data met the criteria of a factor analysis. The principal component factor analysis extracted a three‐factor model namely germane CL, intrinsic CL and extraneous CL, which is exactly the same as the original study. Therefore, we decided to put entire sample size into CFA to confirm the good fit of the three‐factor model. It is worth noting that the *χ^2^/*df, CFI, TLI and RMSEA statistics demonstrated that the three‐factor model offered an acceptable fit with the data collected, indicating that the scale has good construct validity (Hooper, Coughlan, & Mullen, 2008; Kaya et al., 2018). This level of construct validity may benefit the cross‐cultural adaptation of the scale (Kaya et al., 2018) and enable evaluation of different CL in ICU nurses.

Our results are similar to the original study of the English version MDT‐CL (Leppink et al., 2013), CFA in the study by Leppink and colleagues, which indicated that all the goodness‐of‐fit indicators were acceptable, CFI = 0.995, TLI = 0.992 and RMSEA = 0.035. These authors collected data among students in statistics lectures and revealed a three‐factor model (Leppink et al., 2013). We recruited ICU nurses to verify the psychometric properties of the Chinese MDT‐CL. Despite the differences between the study populations and cultural backgrounds, the influence factors of the different CL are the same (Ceballos‐Vasquez et al., 2015; Paas et al., 1994). For example, the difficulty of the activity influences intrinsic CL and the presentation of the activity influences extraneous CL. Therefore, the CFA in our study fully demonstrates that the Chinese version MDT‐CL maintains good construct validity in the tests of ICU nurses.

While there is a lack of a gold standard instrument to measure overall CL and different types of CL, the NASA‐TLX commonly used in China is regarded as the standard to confirm the Chinese version MDT‐CL (Xiao et al., 2005). In addition, Paas's nine‐point scale, which is commonly used in China, is regarded as the standard to measure overall CL. Besides that, Ayres, Cierniak and Salomon developed three different single items to measure intrinsic, extraneous and germane CL, respectively. Significant positive correlation between the measures was found, which indicates good criterion validity of the MDT‐CL. However, the correlation coefficients were not good between the NASA‐TLX and extraneous CL and germane CL (*r* = 0.495, 0.564, respectively). The possible reason is that NASA‐TLX was more appropriate as a standard instrument to assess overall CL, but not for intrinsic, extraneous and germane CL (Sonmez et al., 2017). Therefore, we used three single items as standard instrument to measure three different types of CL.

Good correlation coefficients were found between each factor and the overall MDT‐CL scale. Our results are in line with the original English version (Leppink et al., 2013), which reported that intrinsic CL and germane CL were positively related (*r* = 0.33). Several reasons may help to explain the correlation between intrinsic CL and germane CL. First, if a nursing care activity is too easy for a nurse (low intrinsic CL), an explanation on how to perform the activity in the process is unnecessary and add extraneous CL to the nurse. Therefore, few cognitive resources are allocated to germane CL (Hsieh et al., 2016). Second, if a nursing care activity is too complex for a nurse (high intrinsic CL), cognitive capacity for germane CL activities may be limited. Extremely low or high levels of intrinsic CL may limit germane CL activity (Hsieh et al., 2016). Therefore, a weak positive correlation is expected between intrinsic CL and germane CL. The results of our study further confirm the positive correlation between the intrinsic CL and germane CL. However, the correlation between extraneous CL and germane CL (*r* = 0.003) in our study is different than the original study (*r *= −0.19). If a nursing care activity is presented to a nurse in a simple or familiar way, and there is a lower extraneous CL, it seems that the nurse may devote more cognitive resources to germane CL activities (Dal Sasso & Barra, 2015; Hsieh et al., 2016). Therefore, extraneous CL and germane CL are expected to be negatively correlated. The results of our study do not show a negative correlation between extraneous CL and germane CL. This result may be explained by the fact that in our study hospital managers treated ICU delirium with indifference, and a disregard for the problem of ICU delirium by nurse managers resulted in a lack of nurses’ knowledge of ICU delirium (Hickin, White, & Knopp‐Sihota, 2017; Rowley‐Conwy, 2018). For a nurse with lower delirium knowledge, even if an activity has a low extraneous CL, he or she still cannot devote more cognitive resources to germane CL activities due to lack of knowledge. This issue may be the reason why a negative correlation between extraneous CL and germane CL was not demonstrated.

Regarding the test–retest reliability, the extraneous CL was the least stable factor among the three identified factors in the Chinese version MDT‐CL. The possible reason might include the following: (a) there are only three items included in the extraneous CL and Leppink et al. (Leppink et al., 2013) indicated that the stability of a scale or subscale can be affected when there are only few items within a scale or subscale and (b) extraneous CL may vary when the participant's emotions (Pawar et al., 2018) or the physical environment (Aldekhyl, Cavalcanti, & Naismith, 2018) is subject to change. Future studies are needed to conduct equivalence reliability for testing the stability of the extraneous CL (Park et al., 2018).

There is thus a compelling need for hospital managers to assess the CL of health care workers, and in this study, the MDT‐CL was translated into Chinese, and the reliability and validity of its clinical application were preliminarily evaluated with the attainment of satisfactory results. The validated Chinese version scale in this study is supported for use in clinical settings to evaluate nurses for the presence of high CL, in particular to monitor different types of CL which are caused by a variety of reasons and assess the effectiveness of interventions for reducing different types of CL (Duhoux et al., 2017). Another important implication is that this study provides a new method for discussing nurses' CL. This study also provides scientific theoretical basis and research direction for the further development of CLT in nursing research.

## 6 LIMITATIONS

Several limitations of the study should be noted. First, a convenience sample was used in only three university‐affiliated hospitals in the north of China, and also, a relatively small number of males were included in our study, which may limit representativeness of the sample and the generalization of the results. Nevertheless, the total sample size meets the requirement for CFA. Future studies should enlarge the sample size and target diverse departments in hospitals in different regions of the country, to further examine whether the Chinese version MDT‐CL is suitable for use in Chinese culture. Second, we investigated only construct validity, criterion validity and internal consistency of the MDT‐CL scale. Future studies should focus on the predictive validity of the scale. Despite these limitations, our study demonstrates that the Chinese version MDT‐CL is a reliable and valid instrument which can be used to evaluate the different types of CL among hospital medical workers in Mainland China.

## 7 CONCLUSIONS

This study evaluated MDT‐CL among Chinese nurses and demonstrated satisfactory psychometric properties with good reliability and validity when applied to measure different types of CL. Administrators may use the MDT‐CL as an appropriate and effective method to assess the three different types of CL of health care workers and to identify individuals who are prone to high CL. This assessment can facilitate tailored individual interventions to reduce specific types of CL among clinical staff and improve the quality of patient care.

## CONFLICT OF INTEREST

We have no conflicts of interest to declare.
