# Supplementary material for: Psychometric properties of the Chinese version of the instrument for measuring different types of cognitive load (MDT‐CL)
Source: J Nurs Manag. 2020 Mar 28;28(2):277–85. doi: 10.1111/jonm.12919 (PMC7161924; doi:10.1111/jonm.12919)
Supplement: Supplementary file 1 [file JONM-28-277-s001.docx]

**Original English version of the instrument for Measuring Different Types of Cognitive Load (MDT-CL)**

A ten-item questionnaire for the measurement of intrinsic cognitive load (Items 1, 2, and 3), extraneous cognitive load (Items 4, 5, and 6), and germane cognitive load (Items 7, 8, 9, and 10)

All of the following questions refer to the activity (lecture, class, discussion session, skills training or study session) that just finished. Please respond to each of the questions on the following scale (0 meaning not at all the case and 10 meaning completely the case).

0 1 2 3 4 5 6 7 8 9 10

[1] The topic/topics covered in the activity was/were very complex.

[2] The activity covered formulas that I perceived as very complex.

[3] The activity covered concepts and definitions that I perceived as very complex.

[4] The instructions and/or explanations during the activity were very unclear.

[5] The instructions and/or explanations were, in terms of learning, very ineffective.

[6] The instructions and/or explanations were full of unclear language.

[7] The activity really enhanced my understanding of the topic(s) covered.

[8] The activity really enhanced my knowledge and understanding of statistics.

[9] The activity really enhanced my understanding of the formulas covered.

[10] The activity really enhanced my understanding of concepts and definitions.

**测量三种不同认知负荷的量表（MDT-CL）**

以下所有问题都涉及到日常护理工作中的谵妄照护任务（谵妄评估、危险因素评估、预防及干预措施实施）。请回答以下每一个问题，从0-10打分(0表示完全不同意，10表示完全同意)。

1.日常护理工作中的谵妄照护任务非常复杂。

2.日常护理工作中的谵妄预防处理措施，我认为非常复杂。

3.日常护理工作中的谵妄及危险因素评估，我认为非常复杂。

4.在日常护理工作中实施谵妄照护任务时，我认为指导说明/解释非常不清楚。

5.日常护理工作中谵妄照护任务的指导说明/解释在临床应用方面是非常无效的。

6.日常护理工作中谵妄照护任务的指导说明/解释存在很多表达不清楚的语言。

7.日常护理工作确实增进了我对谵妄照护任务的理解。

8.日常护理工作确实提高了我对谵妄的认识和理解。

9.日常护理工作确实增进了我对谵妄预防处理措施的理解。

10.日常护理工作确实增进了我对谵妄及危险因素评估的理解。

**Chinese version of the instrument for measuring different types of cognitive load**

A ten-item questionnaire for the measurement of IL (Items 1, 2, and 3), EL (Items 4, 5, and 6), and GL (Items 7, 8, 9, and 10). All of the following questions refer to the activity (delirium diagnosis, assessment of risk factors, implementation of delirium prevention and management interventions) that just finished. Please respond to each of the questions on the following scale (0 meaning not at all the case and 10 meaning completely the case).

1. The delirium care activity/activities presented in daily usual care was/were very complex.
2. The delirium prevention or management interventions presented in daily usual care that I perceived as very complex.
3. The delirium and risk factors assessment presented in daily usual care that I perceived as very complex.
4. The instructions and/or explanations in daily usual care during implementing delirium care activity/activities were very unclear.

[5] The instructions and/or explanations in daily usual care were, in terms of clinical application, very ineffective.

[6] The instructions and/or explanations in daily usual care were full of unclear language.

[7] Daily usual care really enhanced my understanding of the delirium care activity/activities covered.

[8] Daily usual care really enhanced my knowledge and understanding of delirium.

[9] Daily usual care really enhanced my understanding of the delirium prevention or management interventions.

[10] Daily usual care really enhanced my understanding of delirium and risk factors assessment.

Copyright **©**School of Nursing, Capital Medical University/Shan Zhang, Ying Wu: translated with kind permission of Maastricht University in the Netherlands /Jimmie Leppink.
